# BET inhibitors rescue anti-PD1 resistance by enhancing TCF7 accessibility in leukemia-derived terminally exhausted CD8^+^ T cells

**DOI:** 10.1038/s41375-023-01808-0

**Published:** 2023-01-21

**Authors:** Kyle A. Romine, Kevin MacPherson, Hyun-jun Cho, Yoko Kosaka, Patrick A. Flynn, Kaelan H. Byrd, Jesse L. Coy, Matthew T. Newman, Ravina Pandita, Christopher P. Loo, Jaime Scott, Andrew C. Adey, Evan F. Lind

**Affiliations:** 1 Department of Cell, Developmental & Cancer Biology, Oregon Health & Science University, Portland, OR USA; 2 Department of Molecular & Medical Genetics, Oregon Health & Science University, Portland, OR USA; 3 Department of Molecular Microbiology and Immunology, Oregon Health & Science University, Portland, OR USA; 4 School of Medicine, Oregon Health & Science University, Portland, OR USA; 5 Knight Cancer Institute, Oregon Health & Science University, Portland, OR USA; 6 Knight Cardiovascular Institute, Oregon Health & Science University, Portland, OR USA; 7 Center for Early Detection Advanced Research, Oregon Health & Science University, Portland, OR USA

**Keywords:** Tumour immunology, Cancer models

## Abstract

Many acute myeloid leukemia (AML) patients exhibit hallmarks of immune exhaustion, such as increased myeloid-derived suppressor cells, suppressive regulatory T cells and dysfunctional T cells. Similarly, we have identified the same immune-related features, including exhausted CD8^+^ T cells (TEx) in a mouse model of AML. Here we show that inhibitors that target bromodomain and extra-terminal domain (BET) proteins affect tumor-intrinsic factors but also rescue T cell exhaustion and ICB resistance. Ex vivo treatment of cells from AML mice and AML patients with BET inhibitors (BETi) reversed CD8^+^ T cell exhaustion by restoring proliferative capacity and expansion of the more functional precursor-exhausted T cells. This reversal was enhanced by combined BETi and anti-PD1 treatment. BETi synergized with anti-PD1 in vivo, resulting in the reduction of circulating leukemia cells, enrichment of CD8^+^ T cells in the bone marrow, and increase in expression of *Tcf7*, *Slamf6*, and *Cxcr5* in CD8^+^ T cells. Finally, we profiled the epigenomes of in vivo JQ1-treated AML-derived CD8^+^ T cells by single-cell ATAC-seq and found that JQ1 increases *Tcf7* accessibility specifically in Tex cells, suggesting that BETi likely acts mechanistically by relieving repression of progenitor programs in Tex CD8^+^ T cells and maintaining a pool of anti-PD1 responsive CD8^+^ T cells.

## Introduction

Acute myeloid leukemia (AML) is a genetically heterogeneous myeloid lineage cancer with a 5-year survival of 29% and with limited therapeutic options for those who cannot withstand current frontline therapies [1–3]. The most common mutation, which encompasses 30% of AML patients and is associated with an exceptionally poor prognosis, is an internal tandem duplication in the FLT3 receptor (FLT3-ITD), which leads to ligand-independent signaling to many proliferation pathways [4]. FLT3-ITD is commonly mutated with epigenetic regulators such as those involved in DNA methylation like DNMT3a and TET2 [5]. For other difficult-to-treat cancers, such as metastatic melanoma, therapy with immune checkpoint blockade (ICB) has made meaningful increases in life expectancy [6, 7]. ICB functions to reinvigorate cancer-specific T cells via blockade of inhibitory immune checkpoint (IC) receptors that suppress T cell activity and function. Expression of IC receptors, which include CTLA-4, PD1, TIM3, LAG3, TIGIT, VISTA, and others, increases after initial antigen exposure and regulates T cell function through various signaling pathways. It has been hypothesized that these receptors are an evolutionary adaptation to chronic antigen exposure to prevent the development of autoimmunity. Recent work has identified unique subsets of CD8^+^ T cells that are generated specifically during chronic antigen exposure. Terminally exhausted T cells (TEx) have been shown to express high levels of IC receptors such as PD1 and TIM3 and low TCF1^,^ while progenitor exhausted T cells (TPEx) are PD1^+^, TIM3^-^ and display high expression of TCF1 and SLAMF6. Importantly, the TPEx population has specifically been shown to expand in response to anti-PD1 therapy and retain anti-tumor capacity [8–12]. In contrast, TEx are significantly more dysfunctional with a lack of proliferative capacity and dramatically reduced secretion of effector molecules, such as granzyme B and perforin [8–10, 13–16]. Our lab and others have recently shown that a proportion of blood and bone marrow specimens from AML patients have an immunosuppressive microenvironment and have hallmarks of immune exhaustion: increased frequencies of regulatory T cells (Tregs) [17] and myeloid-derived suppressor cells (MDSCs) [18], decreased T cell proliferation [19], elevated expression of IC molecules [19–21] and increased TEx vs. TPEx [21, 22] populations. Importantly, a subset of these patient samples containing dysfunctional T cells can be rescued by ICB [19]. This prompted us to further investigate potential combination treatment regimens by which immune exhaustion could be reduced in AML.

The bromodomain and extra-terminal domain (BET) protein family is made up of bromodomain-containing proteins BRD2, BRD3, BRD4, and BRDT [23–26]. BET proteins are epigenetic readers which bind acetylated histone residues via conserved BD1 and BD2 domains and mediate downstream functions, such as histone acetylation recognition, chromatin remodeling, and transcription regulation. BRD4 binds acetylated histone tails and recruits Positive Transcription Elongation Factor b to enhancer regions to mediate the phosphorylation of the c-terminal domain of RNA polymerase II, required for elongation of the mRNA strand [24, 27]. Loss of function RNAi screening studies identified BRD4 loss as a potent and selective inhibitor of leukemic growth, and treatment with BET inhibitor (BETi) induced leukemia cell death in vitro and in vivo [23, 25, 28, 29]. Clinically, however, BETi therapy was able to elicit some complete remissions but only in a small subset of patients [30]. Interestingly, BETi have also been shown to positively affect CD8^+^ T cells, directly or indirectly, via reduction of PDL1 expression on myeloid cells [31, 32] and inhibition of chronic T cell receptor (TCR) activation genes, such as basic leucine zipper transcription factor, ATF-like (*Batf*), which increased the persistence of stem-cell-like memory CD8^+^ T cells [33]. Therefore, we hypothesized that BETi may synergize with anti-PD1 therapy in AML through targeting of tumor-intrinsic factors, such as *myc*, and tumor-extrinsic factors, such as promoting T cell stemness. Accordingly, we showed that a *LysM- Cre Flt3-ITD/Tet2* AML mouse model, similar to the previously described *Vav-Cre Flt3-ITD/Tet2* model [34] but retaining wild-type T cells, phenocopies the immune exhaustion and PD1 refractoriness found in many patients with AML. In addition, these mice had increased clonal diversity, as determined by TCR-seq, indicating that the effects are driven by endogenous leukemia antigens. We found that these mice have a dramatically increased ratio of TEx:TPEx compared to wild-type (WT) mice and that BETi can rescue immune exhaustion by promoting the proliferation of TPEx CD8^+^ T cells and reprogramming Tex cells. BETi treatment was found to have a synergistic effect with anti-PD1 in vivo and ex vivo by reducing leukemic tumor burden and increasing RNA expression of TPEx gene programs while decreasing that of TEx. In addition to the mouse model, we observed that BETi rescued T cell exhaustion in a subset of primary AML patient samples. Finally, we characterized the chromatin landscape of T cells treated in vivo with BETi by S3-ATAC-seq and found that BETi-treated Tex had greater *Tcf7* accessibility compared to vehicle-treated throughout TPEx to TEx differentiation.

## Methods

### AML murine model

Mice expressing FLT3-ITD under the endogenous *Flt3* promoter (strain B6.129-Flt3tm1Dgg/J, The Jackson Laboratory, stock no. 011112) [35] were crossed to mice with the *Tet2* gene flanked by LoxP sites (strain B6;129S-Tet2tm1.1Iaai/J, The Jackson Laboratory, stock no. 017573) [36]. The *Flt3-ITD/Tet2* ^*flox*^ mice were then crossed to mice expressing Cre recombinase under the *Lys*m promoter (strain B6.129P2-Lyz2tm1(cre)Ifo/J, The Jackson Laboratory, stock no. 004781). All breeding animals were purchased from The Jackson Laboratory. All mice used in these experiments were bred as heterozygous for FLT3-ITD, TET2, and LysCre. All mouse experiments were performed in accordance with the OHSU Institutional Animal Care and Use Committee protocol IP00000907. No inclusion or exclusion criteria were used on the animals with correct genotype. Mice were selected and assigned to groups randomly while maintaining a 50% male 50% female ratio per experiment. No blinding was performed. Average age of mice used for in vivo studies was 35 weeks. In order to detect a statistical difference of 50%, with approximately 30% standard deviation, powered to 0.8, alpha of 0.05, a sample size of seven mice per experimental group was used.

### Flow cytometry staining

Bone marrow, blood, or splenocytes were processed and subjected to red blood cell lysis by ACK before counting via hemacytometer. Five million cells were resuspended in PBS and stained with Zombie Aqua viability dye (BioLegend, Cat# 423102) for 15 min at room temperature, covered from light. The cells were then washed with FACS buffer (PBS, 2% calf serum, 0.02% sodium azide) and resuspended in 25 µL 1:50 mouse FC block (TruStain FcX, BioLegend Cat# 101320), and left on ice for 5 min. 25 µL of a 2× cell surface staining antibody cocktail was added directly on top of the cells (final FC block 1:100, 1× Ab concentration) and stained on ice for 30 min. For intracellular staining, the cells were then washed with FACS buffer, permeabilized, and stained for intracellular targets according to the manufacturer’s protocol (eBioscience FOXP3 Transcription Factor Staining Buffer Set, Cat# 00-5523-00), then resuspended in FACS buffer before analyzing on either a BD Fortessa or Cytek Aurora flow cytometer. Data were analyzed using FlowJo software.

### H&E histology

Liver, spleen, and bone marrow snips were fixed and cut into paraffin blocks for H&E staining. The slides were then scanned using an Aperio AT2 scanner and analyzed with ImageScope.

### Long-term culture of AML mouse-derived bone marrow cells and Inhibitor Library Screen

Bone marrow aspirates from three AML mice were combined, isolated, and cultured in RPMI containing 20% fetal bovine serum (FBS), streptomycin/penicillin, 50 µM 2-mercaptoethanol (RPMI-20), and supplemented with murine SCF (10 ng/mL) and IL-3 (10 ng/mL) (Peprotech or BioLegend). The cells were serially passaged for ~1 month. Inhibitor library screening to evaluate drug sensitivity was performed as previously described [5]. Briefly, cultured AML mouse-derived cells were counted and seeded into four 384-well plates at a concentration of 2000 cells/well. The cells were then subjected to titrations of 188 unique small-molecule inhibitors (SMIs) in culture for 72 h. MTS reagent (CellTiter96 AQueous One; Promega) was added and the optical density was read at 490 nm to assess viability.

### Mouse ex vivo proliferation assays

96-well round-bottom plates were coated with either 2.5 µg/mL anti-CD3 (BioLegend, clone 145-2C11,Cat# 100359) or HIgG (BioLegend, clone HTK888, Cat#400959), at 4 °C overnight, then washed with PBS. Splenocytes were harvested from AML or WT mice and stained with 2 µM CFSE (Life Technologies, Thermo Fisher) at 5 × 10^6^/mL at 37 °C in the dark. After 15 min, the CFSE was quenched with 10 mL calf serum and washed with PBS. Cells were then plated at 5 × 10^5^/well in RPMI with 10% FBS with BME supplementation and penicillin/streptomycin (RPMI-10). After a 72-h incubation at 37 °C the cells were stained for flow cytometric analysis with antibodies listed below and analyzed as previously described. Specificities, clone name and source are listed in Table 1. Anti-PD1 (Clone RMP1-14) and RIgG (2A3) were purchased from BioXCell. JQ1 was purchased from Cayman Chemical (#11187).

**Table 1:** Antibodies used in murine sties and proliferation assays.

Marker	Clone	Vendor
Tbet	4B10	BioLegend
LAG-3	C9B7W	BioLegend
TIM3	RMT3-23	BioLegend
CD44	IM7	BioLegend
CD62L	MEL-14	BioLegend
PDL1	10F.9G2	BioLegend
CD11b	M1/70	BioLegend
CD8a	53-6.7	BioLegend
GR1	RB6-8C5	BioLegend
PD1	29F.1A12	BioLegend
BATF	S39-1060	BioLegend
CD34	HM34	BioLegend
CD3e	400A2	BioLegend
TCF1	C63D9	Cell Signaling Technologies
CD4	GK1.5	BioLegend
FOXP3	FJK-16S	BioLegend

List of specificity, clone and source of each antibody used in mouse flow cytometry experiments.

### Human ex vivo proliferation assays

Bone marrow aspirates and peripheral blood samples were separated by Ficoll density gradient centrifugation. All experiments were performed using freshly isolated cells. Cells were labeled with CellTrace Violet (CTV, Thermo Fisher) and cultured in 96-well plates coated with 1 µg/mL anti-CD3 (BioLegend, Clone UCHT1) or control mIgG (BioLegend, Clone MOPC-21). Groups of wells were then treated with either 10 µg/mL control mIgG, 10 µg/mL anti-PD1 (EH12.2H7), 60 nM JQ1, or 60 nM JQ1 + 10 µg/mL anti-PD1. After 5 days, cells were stained for flow cytometry. Antibody clones and source are listed in Table 2. Viability was determined by Zombie Aqua staining and doublets were gated out of analysis by FSC-A vs. FSC-H. Flow cytometry data were acquired on a BD LSRFortessa or Cytek Aurora and analyzed using FlowJo v10 software. All human sample experiments are approved under IRB protocol #00004422, “Pathogenesis of Acute Leukemia, Lymphoproliferative Disorder and Myeloproliferative Disorders” (PI: Marc Loriaux, MD, PhD). Informed consent was obtained from all patients.

**Table 2:** Antibodies used in this study for staining of patient samples.

Marker	Clone	Vendor
CD19	HIB19	BioLegend
CD45	HI30	BioLegend
CD8	RPA-T8	BioLegend
CTLA-4	BNI3	BioLegend
PDL1	29E.2A3	BioLegend
CD33	WM53	BioLegend
CD4	RPA-T4	BioLegend
PDL2	24F.10C12	BioLegend
CD56	HCD56	BioLegend
TIGIT	MBSA43	Thermo Fisher
TIM3	7D3	BD
PD1	EH12.2H7	BioLegend
CD3	HIT3a	BioLegend

List of specificity, clone and source of each antibody used in human flow cytometry experiments.

### BETi + anti-PD1 treatment in vivo

Age range of AML mice used in in vivo experiments was 25–54 weeks. WT and AML mice were given seven doses of 8 mg/kg RIgG, 50 mg/kg JQ1, 8 mg/kg anti-PD1, or 50 mg/kg JQ1 + 8 mg/kg anti-PD1 via i.p. injection over 2 weeks (3 times per week, harvesting 1 day after 7th/final injection). Drug solutions were prepared in a solvent of 10% cyclodextrin in PBS from a stock solution of JQ1 (50 mg/mL) in DMSO, followed by sonication/heat bath to dissolve the JQ1. Assessment of tumor burden in the blood, as determined by white blood cell (WBC) measurements from Hemavet (950FS, Drew Scientific), was monitored weekly pre-, mid-, and post-treatment. At endpoint, single-cell suspensions of bone marrow, blood, and spleen tissues were stained for flow staining as previously described.

### Evaluation of CD8^+^ T cell transcripts via Nanostring

AML mice were given seven doses of vehicle (10% cyclodextrin) *n* = 5 or 50 mg/kg JQ1, *n* = 5, via i.p. injection over 2 weeks (3 times per week, harvesting 1 day after 7th/final injection) as previously. CD8^+^ T cells were isolated by magnetic bead isolation (BioLegend #480136) and RNA was extracted according to the manufacturer’s protocol (PureLink RNA kit, Thermo Fisher). Samples were hybridized and loaded onto the Nanostring Chipset according to the manufacturer’s instructions (Nanostring Panel NS_MM_CANCERIMM_C3400) and analyzed using the nSOLVER4.0 tool.

### s3-ATAC-seq methodology and analysis

AML mouse CD8^+^ T cells were enriched with one round of mojosort magnetic bead sort as described previously. Cells were then prepared as described in Mulqueen et al. [37], with the exception that the tagmentation temperature was reduced to 42 °C as opposed to 55 °C to achieve a higher TSS enrichment on immune cells vs. brain cells for which the initial protocol was developed. S3-ATAC-seq libraries were then sequenced on a NextSeq 2000 instrument and demultiplexed as in Mulqueen et al. [37] prior to conversion into fragment files. Data were then analyzed and visualized using the ArchR package [38]. These data have been deposited under the GSE Accession: GSE205386.

In brief, we analyzed this data using the ArchR pipeline. Samples were concatenated into one file and reformatted into an arrow file and annotated using the mm10 mouse genome. Quality control metrics and doublet removal were performed as described in the ArchR package using the doubletscore and filterdoublets functions. Gene score matrices were generated using the native GeneScoreMatrix function in ArchR. The Peak matrix was generated using macs2. Pseudotime trajectories were generated using the native ArchR trajectory function. The path was chosen based on characteristic gene scores for naïve, TPEx, and TEx as detailed in the corresponding figure. Code available upon request.

### TCR-sequencing via adaptive

Genomic DNA was extracted using a Qiagen DNeasy kit from splenocytes derived from five WT and five AML mice. TCR beta sequencing was then performed using immunoSEQ from Adaptive Biotechnologies. The data were then analyzed using the immunoSEQ Analyzer Tool.

### Statistical analysis

Statistical analysis was carried out in GraphPad Prism software V9.2.0. Analysis consisted of Mann–Whitney *t*-test, Kruskal–Wallis multiple comparisons *t*-tests or one-way ANOVA. *P* values of less than 0.05 were considered statistically significant. Error bars represent standard error of the mean.

## Results

### *Flt3-ITD/Tet2* mice exhibit hallmarks of immune exhaustion

**Fig. 1:**
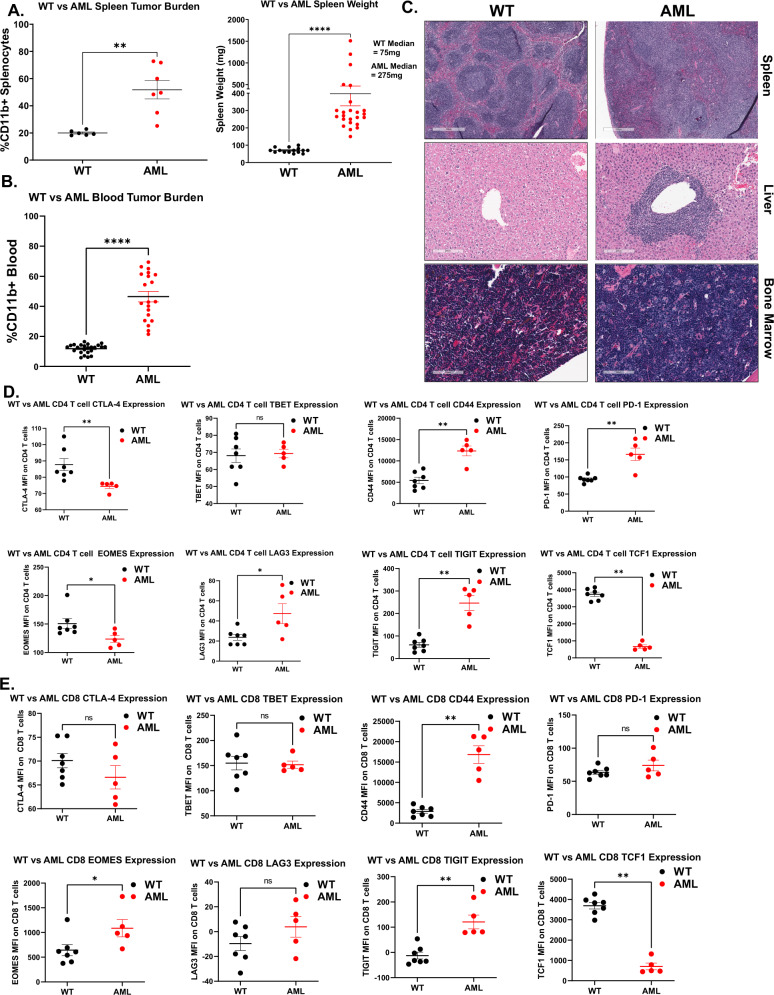
GEMM mouse model characterization. **A** Left: splenocytes isolated from 6 C57BL/6 (WT, black) or 7 *Flt3-ITD*+*/–, Tet2*+*/–, Lys-Cre*+*/–* (AML, red) mice were stained for live CD11b+ cells and evaluated by flow cytometry. Significance determined by Mann–Whitney *t*-test. Right: whole spleens isolated from 15 WT (black) and 24 AML (red) mice were weighed. Median weights for each group are displayed on the right side of plot. Significance determined by Mann–Whitney *t*-test. **B** Blood isolated from 24 WT (black) and 20 AML (red) mice were stained for live CD11b+ cells and evaluated by flow cytometry. Significance determined by Mann–Whitney *t*-test. **C** Representative H&E staining of WT and AML mice-derived spleen (top row, 500 µm scale), liver (middle row, 200 µm scale), and bone marrow (bottom row, 100 µm scale). **D** Splenocytes from untreated 7 WT (black) and 5 AML (red) mice were assessed for expression (median fluorescence intensity) of markers of immune exhaustion on CD4^+^ T cells (Live, CD11b-, CD3^+^, CD4^+^). Top row, left to right: CTLA-4, TBET, CD44, PD1). Bottom Row, left to right: EOMES, LAG3, TIGIT, TCF1). Significance determined by multiple Mann–Whitney *t*-tests. CD44, PD1, LAG3 are significantly increased in AML mice. CTLA-4, EOMES, and TCF1 are significantly decreased in AML mice. **E** Splenocytes from untreated WT (black) and AML (red) mice were assessed for expression (median fluorescence intensity) of markers of immune exhaustion on CD8^+^ T cells, as in (**D**). Significance determined by multiple Mann–Whitney *t*-tests. CD44, EOMES, and TIGIT are significantly increased in AML mice.

We previously showed that a genetically engineered mouse model, *Flt3-ITD*^*+/–*^ *Tet2*^*flox/+*^ *LysM-Cre*^*+/–*^ displays a profound defect in T cell proliferative capacity [39]. To further characterize the tumor microenvironment and functional capacity of T cells in these mice, we assessed changes in myeloid and lymphoid populations at multiple organ sites. We found significantly increased levels of CD11b^+^ myeloid cells in the spleens and blood of AML mice compared to WT mice (Fig. 1A, B). We observed significant evidence of myeloid infiltration in the spleen causing splenic follicle disruption as well as infiltration in both the liver portal veins and bone marrow (Fig. 1C). Furthermore, we found increased levels of GR1^+^ MDSCs and CD4^+^FOXP3^+^ Tregs, a frequent occurrence in AML patients [17–20, 40] (Supplementary Fig S1A, B), but no difference in T cell frequency (Supplementary Fig S1C). In addition, there were no significant differences in the ratio of CD4:CD8 T cells in AML mice (Supplementary Fig S1D). We also identified significant increases in multiple exhaustion markers in both the CD4^+^ and CD8^+^ T cell populations but most notably a significant decrease in T cell stem-like maintenance transcription factor TCF1 (Fig. 1D, E). Taken together, we concluded that these AML mice exhibited a tumor microenvironment characterized by immune activation.

### T cells derived from AML mice are clonally expanded as well as phenotypically and functionally exhausted

**Fig. 2:**
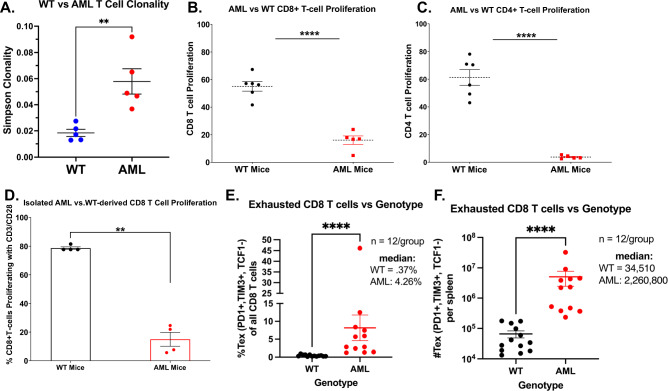
AML mouse-derived CD8^+^ T cells are intrinsically dysfunctional and unresponsive to TCR stimulation. **A** Genomic DNA was extracted from 5 WT and 5 AML mouse splenocytes and sequenced for TCRb. Simpsons Clonality was calculated and plotted (WT mice in black, AML mice in red). Significance determined by Mann–Whitney *t*-test. **B**, **C** 6 WT (black) and 5 AML (red) splenocytes were isolated, stained with proliferation dye (CFSE), and cultured for 72 h with anti-CD3. Proliferation of **A** CD8^+^ T cells and **B** CD4^+^ T cells was then assessed by flow cytometry, staining with viability and markers to identify T cells. Proliferation displayed is percent CFSE diluted relative to unstimulated (HIgG) control for each cell type. Significance determined by Mann–Whitney *t*-tests. **D** 4 WT (black) and 4 AML (red) derived T cells were isolated from splenocytes via CD3 negative isolation magnetic beads. The T cells were then stained with CFSE and plated for 72 h with anti-CD3 and anti-CD28 stimulation. The cells were then harvested and assessed by flow cytometry. Significance determined by Mann–Whitney *t*-test. **E**, **F** Splenocytes derived from 12 WT (black) and 12 AML (red) mice were stained for surface and intracellular markers of T cell exhaustion and evaluated by flow cytometry. Terminally Exhausted CD8 T cells (TEx) are represented as a **E** fraction of all CD8 T cells (%PD1+, TIM3+, TCF1-) and **F** calculated total number of cells per spleen. Significance was determined by Mann–Whitney *t*-tests.

To determine whether AML-derived T cells were clonally responding to leukemia antigens, we performed TCRb sequencing and measured TCR diversity at baseline in WT and AML mice. We found that AML mice had significantly increased Simpson Diversity compared to WT T cells, indicating that they are recognizing leukemia antigens (Fig. 2A). We previously showed that T cells from the AML mice are dysfunctional and unresponsive to TCR stimulation [39]. We assessed the proliferative potential of CD4^+^ and CD8^+^ T cells derived from AML or WT mice by culturing whole splenocytes on anti-CD3 coated plates for 3 days, thus relying on naturally occurring antigen-presenting cells for co-stimulation. As shown previously, both CD4^+^ and CD8^+^ T cells derived from AML mice exhibited significantly reduced proliferation compared to WT mice in response to TCR stimulation (Fig. 2B, C). We next asked whether the T cells were intrinsically dysfunctional or if the effect on proliferation occurred only when the T cells were in proximity to tumor cells. We isolated CD3^+^ T cells by negative selection magnetic bead sorting and assessed proliferation with both anti-CD3 and anti-CD28 co-stimulation. The purified T cells from AML mice still lacked proliferative capacity compared to WT, indicating that the T cells are intrinsically dysfunctional (Fig. 2D). Finally, we asked whether the AML-derived CD8^+^ T cells were phenotypically terminally exhausted (TEx; PD1^+^, TIM3^+^, TCF1^-^) or TPEx (PD1^+^, TCF1^+^, TIM3^-^), the latter having been shown to retain anti-tumor activity, predict clinical outcome, and expand with ICB [9, 11, 41–44]. Flow cytometric analysis of CD8^+^ T cells derived from AML mice was primarily consistent with a TEx phenotype, in contrast to WT mice (Fig. 2E and Supplementary Fig. S2A). Thus, the AML mice generate leukemia antigens that induce an immunosuppressive microenvironment that supports CD8^+^ and CD4^+^ T cell exhaustion.

### Ex vivo treatment of splenocytes from AML mice with BETi rescues T cell dysfunction

**Fig. 3:**
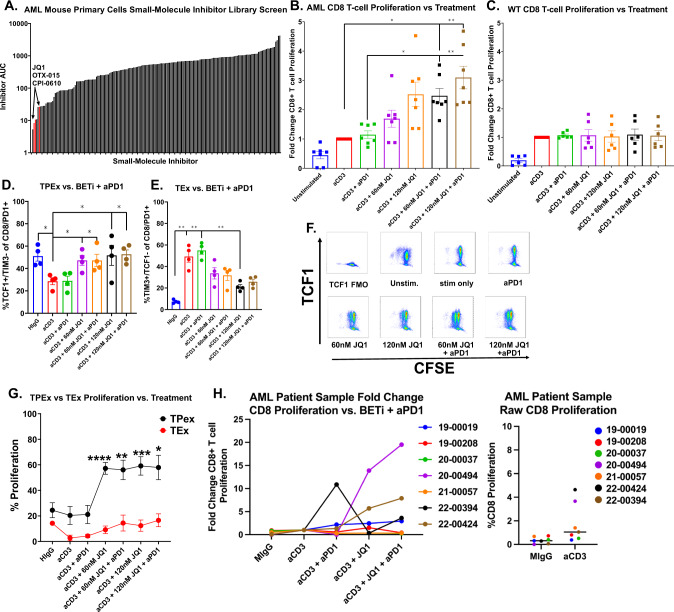
AML mouse T cells are refractory to ICB therapy but partially rescued with BET inhibition. **A** Cells from AML mice passaged for ~1 month were subjected to an inhibitor library panel, as previously described [5]. Cells were seeded into multiple 384-well plates containing titrations of 188 inhibitors, incubated for 72 h, and viability assessed by MTS assay. Plot represents each areas under the curve (AUC) for every inhibitor on the panel. BET inhibitors JQ1, OTX-015, and CPI-0610 are highlighted in red. **B**, **C** Splenocytes were isolated from 7 AML (**B**) or 6 WT mice (**C**), stained with CFSE, and cultured for 72 h without TCR stimulation (HIgG), anti-CD3 alone, anti-CD3 with anti-PD1 or titrations of anti-CD3 with JQ1 or anti-CD3 with both JQ1 and anti-PD1. Cells were stained and analyzed by flow cytometry. Plots represent the fold-change in proliferation in CD8^+^ T cells, as measured by percent CFSE diluted relative to anti-CD3 stimulated alone. Significance determined by Kruskal–Wallis multiple comparisons *t*-tests. **D**, **E** Effect of BETi, anti-PD1, or BETi + anti-PD1 treatment on the percent of TPEx CD8^+^ T cells (**D**) and TEx CD8^+^ T cells (**E**) after culturing for 72 h as previously in **B** and **C**. Significance determined by Kruskal–Wallis multiple comparisons *t*-tests. **F** Representative dot plots showing the proliferation of CD8^+^ T cells vs. TCF1 expression from an AML mouse proliferation assays as described in **B** and **C**. Each panel shows a different condition. X-axis denotes proliferation dye CFSE. Y-axis denotes TCF1 expression. **G** Splenocytes from 4 AML mice were stained with CFSE and plated for 72 h without TCR stimulation (HIgG), anti-CD3 alone, anti-CD3 with anti-PD1 and titrations of anti-CD3 with JQ1 or anti-CD3 with both JQ1 and anti-PD1. Proliferation of TPEx (black) and TEx (red) CD8^+^ T cells from AML mice was assessed by flow cytometry. Significance determined by Kruskal–Wallis multiple comparisons *t*-tests. **H** Fresh mononuclear cells from bone marrow aspirates or peripheral blood obtained from 7 patients with AML were stained with CTV and cultured for 5 days without TCR stimulation (mIgG), anti-CD3, anti-CD3 with anti-PD1, anti-CD3 with 120 nM JQ1 or anti-CD3 with 120 nM JQ1 and anti-PD1. Cells were stained and analyzed by flow cytometry. Left panel plot represents the fold-change CD8^+^ T cell proliferation for each condition vs. respective anti-CD3 alone control for each patient sample. Right panel plots the actual percent proliferation for each AML patient sample with MIgG or CD3 alone.

To identify rationally derived combination treatment strategies which target tumor-intrinsic and extrinsic factors, we performed high-capacity drug screening as previously described [5] on tumor cells cultured from the AML mice. Interestingly, we found that three of the top six most efficacious SMIs targeted BET proteins (JQ1, OTX-015, CPI-0610) (Fig. 3A). Given previous works by Kagoya et al., Zhu et al., and Hogg et al. which established the role for BETi in maintaining stemness of CD8^+^ T cells via inhibition of TCR-activated transcription factor BATF [33] and that BETi reduce immunosuppressive ligand PDL1 [31, 32] expression, we asked whether BETi + anti-PD1 could impact the exhausted phenotype found in T cells from our AML mice. To test whether BETi could rescue T cell dysfunction and rescue ICB therapy resistance, we performed proliferation assays as previously described with whole splenocytes and anti-CD3 stimulation but also in the presence of 60 nM BETi JQ1, 120 nM JQ1, 60 nM JQ1 + 10 µg/mL anti-PD1, 120 nM JQ1 + anti-PD1, or anti-PD1 alone. As expected, treatment with anti-PD1 alone had no effect on proliferation, as the T cells were shown to be primarily terminally exhausted (Fig. 2). However, treatment with JQ1 at 60 nM, and more so with 120 nM, significantly increased proliferation of AML-derived CD8^+^ T cells, whereas WT T cells were unaffected. Moreover, the combination of 120 nM JQ1 and anti-PD1 had the largest effect, with a median three-fold increase in CD8^+^ T cell proliferation compared to anti-CD3 stimulation alone (Fig. 3B, C). The benefit of adding BETi with anti-PD1 was also observed in CD4^+^ T cells, but was more variable between individual mice, with some mice only marginally benefitting from combined treatment while others were dramatically enhanced (Supplementary Fig S2B). We next asked what phenotype characterized these newly proliferating CD8^+^ T cells from the AML mice and found that they expressed high levels of TCF1 and low levels of TIM3, indicating that BETi mechanistically acts by increasing TPEx:TEx ratios (Fig. 3D–G). Moreover, we tested the efficacy of combining BETi + anti-PD1 in seven primary AML patient samples with an array of mutational backgrounds. The patient samples did not proliferate in response to TCR stimulation alone, indicating immune cell dysfunction. Two of seven, however, responded to treatment with BETi or BETi + anti-PD1 in both the CD8^+^ and CD4^+^ T cell subsets (Fig. 3H and Supplementary Fig. S2C).

### In vivo-treated AML mice have reduced tumor burden and increased T cell TPEx gene program expression

**Fig. 4:**
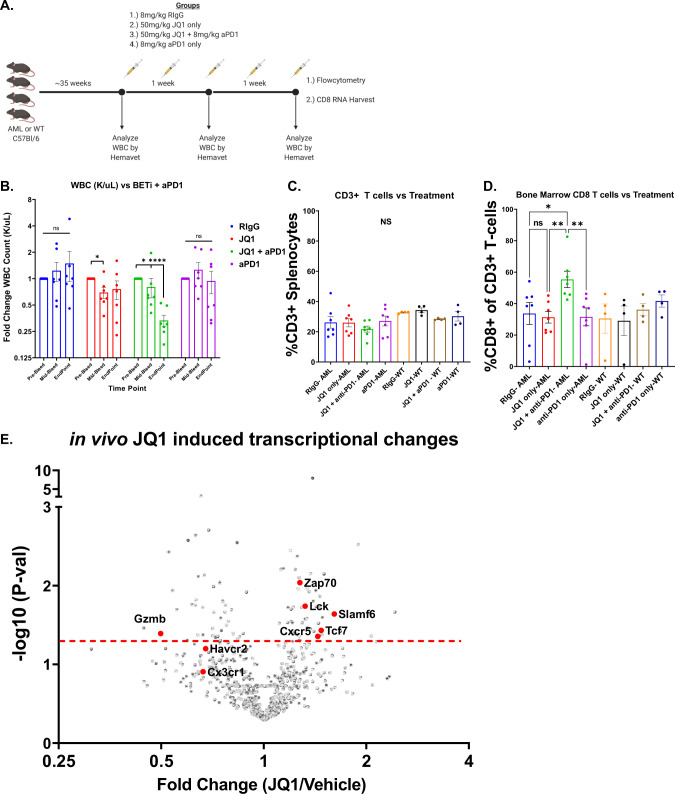
In vivo treatment with BETi and anti-PD1 synergizes in reducing tumor burden and enhances CD8+ T cell activity. **A** Schematic detailing in vivo BETi + anti-PD1 treatment strategy and functional readouts of efficacy. **B** Mice treated with RIgG, JQ1, JQ1 with anti-PD1, or anti-PD1 alone were bled periodically over a 2-week period and assessed for WBC. Data display fold-change WBC (k/µL) normalized per mouse in comparison to pre-treatment bleed WBC. Significance determined by one-way ANOVA. Timepoints are Pre-Bleed, Mid-bleed (day 7), Endpoint (day 14). **C**, **D** Bone marrow cells were isolated from treated AML and WT mice and assessed by flow cytometry. Graph denotes %CD3^+^ T cells in the bone marrow (**C**) and %CD8^+^ T cells in the bone marrow as a percent of all T cells (**D**). Significance derived from combining two experimental replicates and determined by one-way ANOVA. **E** CD8^+^ T cells were isolated from JQ1-treated and RIgG-treated AML mice and RNA harvested and analyzed by Nanostring. Volcano plot shows the fold-change in normalized transcript levels of JQ1-treated mice vs. RIgG-treated AML mice vs. –log_10_ *P* value as determined by multiple *t*-tests with Bonferroni correction. Hits of interest are highlighted in red. Dashed red line denotes significance threshold (0.05).

Given the results showing the effect of BETi on T cells from the AML mice in vitro, we sought to determine the in vivo therapeutic efficacy of BETi in combination with anti-PD1 and whether they also modulate the tumor immune microenvironment. AML or WT mice were treated with RIgG (8 mg/kg), JQ1 (50 mg/kg), anti-PD1 (8 mg/kg), or combined JQ1 and anti-PD1 (50 and 8 mg/kg, respectively) for 14 days. We monitored WBC count in blood pre-treatment, mid-treatment, and at endpoint and characterized the tumor microenvironment (Fig. 4A). Measurement of WBC counts over time by hematology analyzer found that only the combination of JQ1 + anti-PD1 significantly reduced tumor burden and, as expected from the ex vivo proliferation assays, anti-PD1 alone had no effect (Fig. 4B). Characterization of the tumor microenvironment by flow cytometry showed no significant changes to the frequency of Treg cells, MDSCs, or splenic CD3^+^ T cells (Supplementary Fig. S3A, B and Fig. 4C). However, we observed a significant increase in CD8^+^ T cells in the bone marrow of mice in the JQ1 + anti-PD1 treatment group, suggesting that the combination has a greater impact on CD8^+^ T cells specifically and not on other immunosuppressive cell types (Fig. 4D). Finally, evaluation of RNA-transcripts in isolated CD8^+^ T cells derived from JQ1-treated AML mice identified significantly increased expression of TPEx genes such as *Tcf7*, *Slamf6*, and *Cxcr5* (Fig. 4E). Together, these results highlight the potential of combining BETi and anti-PD1 to treat immunosuppressed T cells in AML, particularly in ICB-refractory cases.

### Profiling of the single-cell chromatin landscape of in vivo JQ1-treated T cells with s3-ATAC-seq finds that BETi enhance TCF7 accessibility specifically in Tex cells

**Fig. 5:**
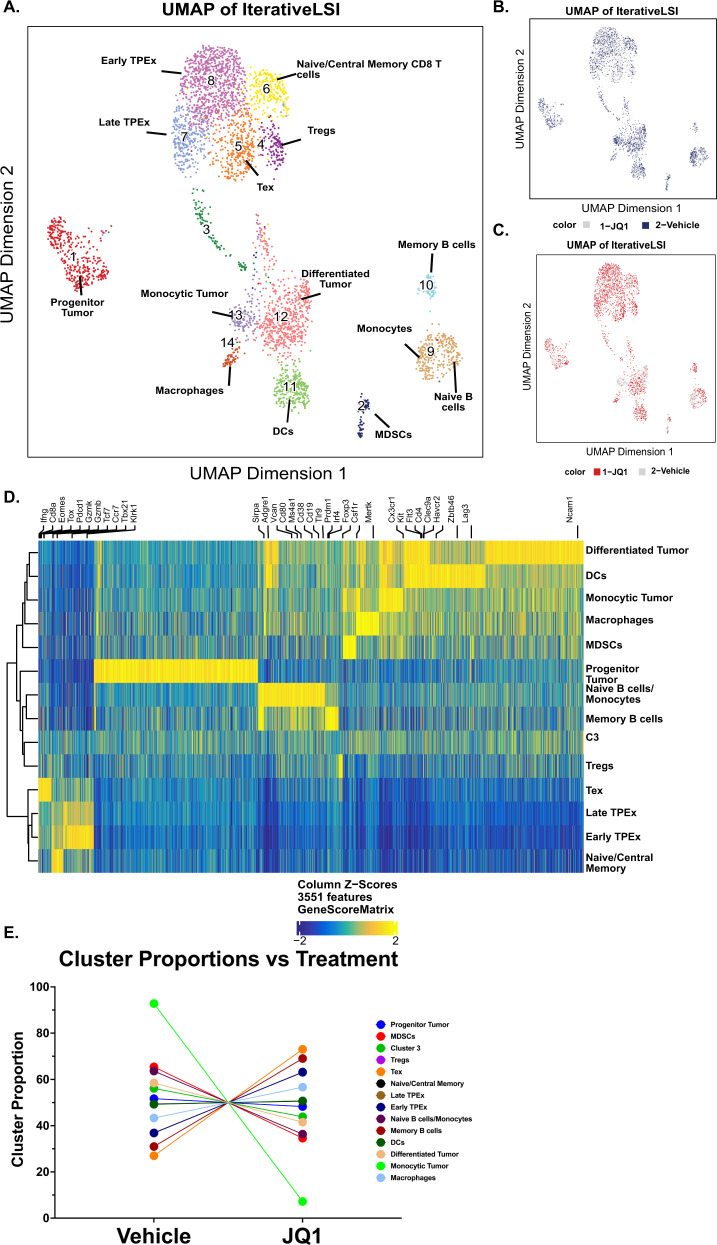
Single-cell chromatin landscape of in vivo BETi-treated CD8^+^ T cells. **A** AML mice were treated in vivo daily for 5 days with vehicle or JQ1 before enriching CD8^+^ T cells via magnetic sort before sequencing via S3-ATAC. Cells were batched and clustered using the ArchR package and cell cluster identities were determined via inferred gene scores of defining gene features. **B**, **C** Overlay of cell proportions based on treatment group. Highlights the distribution of **B** vehicle-treated cells in blue and **C** JQ1-treated cells in red. **D** Heatmap generated from column *Z* scores of significant features for each cluster identified in **A**. Marker genes of interest are highlighted in black text. Cluster identities are described on the right axis. Cells are clustered via Euclidean distance with the corresponding dendrogram drawn on the left axis. **E** Proportions of each cell type were determined as a fraction of all cells in vehicle and JQ1 cells. Each point represents either vehicle-treated (left point) or JQ1-treated (right point) and is connected by a line.

**Fig. 6:**
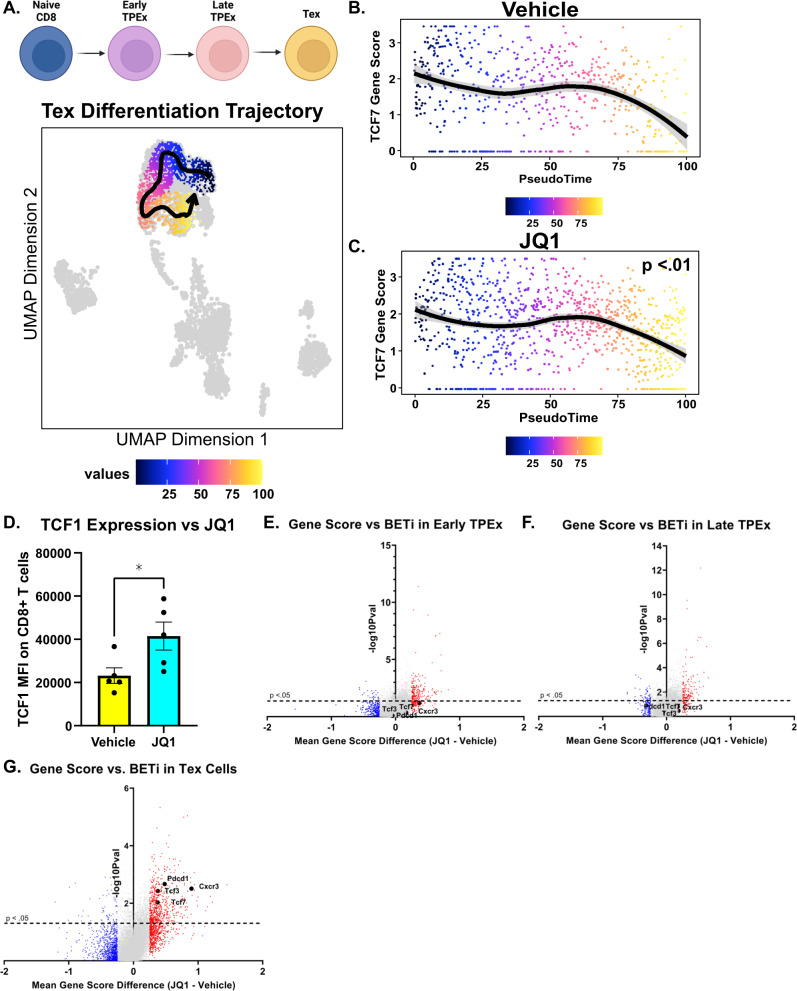
In vivo BETi increases TCF7 accessibility at later stages of TEx differentiation only. **A** (Top) cartoon schematic describing the cell types fit to the differentiation trajectory (bottom). Colors represent pseudotime values throughout TEx differentiation (C6–>C8–>C7–>C5). **B**, **C** Pseudotime trajectory plot with corresponding TCF7 gene score throughout TEx differentiation for **B** Vehicle-treated cells and **C** JQ1-treated cells. Vertical red dashed marks the third quartile throughout pseudotime (late TPex/Early Tex) and the horizontal red dashed line markers the TCF7 accessibility at pseudotime 100. Significance was calculated by Mann–Whitney *t*-test comparing the aggregate TCF7 gene scores for each cell throughout pseudotime in vehicle and JQ1-treated groups. **D** A portion of splenocytes derived from the vehicle or JQ1-treated AML mice were analyzed by flow cytometry. Data represent TCF1 median fluorescence intensity of TCF1 on all CD8^+^ T cells. Significance determined by Mann–Whitney *t*-test. **E**–**G** Volcano plot describing the mean difference in gene scores (JQ1 – Volcano treated) from **E** early TPEx cells only (Cluster 8), **F** late TPEx cells only (Cluster 7), and **G** Tex cells only (Cluster 5). Significance was calculated via multiple corrected *t*-tests. Genes with a mean difference >0.25 are highlighted in red and genes <–0.25 are highlighted in blue. Genes of interest (*Tcf7, Tcf3, Pdcd1*, and *Cxcr3*) are highlighted in black. The dashed horizontal line marks the significance cutoff. Genes below the line are not significant.

To ask whether BETi reprogram Tex directly or block TPex differentiation we evaluated the chromatin landscape of five in vivo JQ1 and five vehicle-treated AML mouse T cells at the single-cell level by s3-ATAC-seq. s3-ATAC-seq is a version of single-cell combinatorial indexed ATAC-seq that achieves a substantial increase in usable reads per cell by leveraging an adapter switching technique to overcome inherent limitations in standard ATAC or other transposase-based workflows [37]. We treated AML mice with vehicle or 50 mg/kg JQ1 daily for 5 days before enriching spleen-derived CD8^+^ T cells by magnetic bead sorting and profiling via s3-ATAC as previously described, with a few optimizations to achieve a cleaner chromatin accessibility signal on immune cell types (methods) [37, 45]. This resulted in a preparation with an aggregate TSS enrichment of 39.31 using ENCODE metrics, making it well above what is considered “ideal” (>7), with ~4000 cells passing QC (Supplementary Fig. S4A, B). Using the R package ArchR [38] we performed iterative latent semantic indexing for dimensionality reduction followed by the identification of 14 distinct clusters (Fig. 5A) distributed between JQ1- and Vehicle-treated splenocytes (Fig. 5B–D). Gene-level accessibility scores were then used to associate each cluster with a cell type based on canonical marker genes (Supplementary Fig S5A). Cluster 4 identified Tregs as denoted by high CD4, FOXP3, and IL2RA gene scores. Clusters 5–8 were CD8^+^ T cells with cluster 5 making up Tex with high *Pdcd1, Tox, Lag3*, and *Havcr2* accessibility. Cluster 6 is denoted as naïve or related CD8^+^ T cells due to their high accessibility at *Sell* and *Tcf7* with low gene scores of any canonical exhaustion or activation markers. Clusters 8 and 7 were assigned as early and late TPex, respectively, due to their high gene scores for *Cxcr3, Tcf7*, and intermediate accessibility at *Pdcd1* and *Sell*. Cluster 8 retained higher gene scores for a number of stem programs also high in cluster 7 but to a lesser degree. Proportionally, JQ1 treatment increased both TEx and TPex, and drastically decreased monocytic tumor cells compared to Vehicle-treated mice (Fig. 5E). The decrease in monocytic tumor cells is consistent with previous works which demonstrated that BETi were exceptionally effective in monocytic leukemia cells [46]. Given our in vivo and ex vivo data demonstrating that JQ1 induces TCF1 expression in CD8^+^ T cells, we investigated *Tcf7* accessibility through pseudotime as Naïve/Stem (C6) differentiate into TPEx (C7/C8) and finally Tex (C5) to ask whether accessibility is affected more in earlier vs. late differentiated T cells (Fig. 6A). Here, we found that JQ1-treated T cells specifically enhance *Tcf7* gene activity in later stages of differentiation as indicated by an elevated gene activity score in late pseudotime (Fig. 6A–C and Supplementary Fig. S6A, B). TCF1 was confirmed to be increased in CD8+ T cells at the protein level by flow cytometry (Fig. 6D). Finally, we calculated the differential gene activity scores in Tex and TPEx clusters and found that there were only significantly increased gene scores for *Tcf7, Cxcr3, Tcf3*, and *Pdcd1* in TEx cells only. While this does not conclusively show that BETi mechanistically act by reprogramming Tex cells directly, it suggests that it may be the primary mechanism (Fig. 6E–G).

## Discussion

### Immune checkpoint blockade in hematological malignancies

ICB therapy in the blood cancer setting is relatively new and although dozens of clinical trials are ongoing and actively recruiting [47], no large-scale study has been completed evaluating the efficacy of anti-PD1 therapies in AML. Our lab previously described the functional immune microenvironment of 50 newly diagnosed AML patient bone marrow samples [19] and found that only 41% showed CD8^+^ T cell proliferative capacity that was comparable to healthy donors. Furthermore, we found that the AML patient samples with the most profound proliferative defect (37% of those tested) had severely dysfunctional T cells with significantly decreased production of effector cytokines such as IFN-γ, IL-2, and TNF-α, and increased expression of inhibitory checkpoint markers such as CTLA-4. Of these non-proliferating AML samples, 6 of 18 samples were not rescued with ICB treatment in vitro, maintaining the defects in proliferation and effector molecules upregulation seen without ICB. However, 12 of 18 samples did respond, at least partially, to ICB indicating that suppressive mechanisms may be overcome by altering the capacity of T cells to receive inhibitory signals through IC in the tumor environment. Combination treatment regimens that target both the tumor and immune environment to rescue terminally TEx may be the key to greater success with ICB therapy clinically. Clinical trials have evaluated the efficacy of anti-PD1+ hypomethylating agents (HMA) but only a fraction responded to therapy [48, 49], which is consistent with our ex vivo findings [19]. Phenotypic profiling of these responsive patients before and after anti-PD1+/– HMA identified pre-therapy bone marrow and peripheral blood CD3^+^ and CD8^+^ levels as predictive of response. In addition, we previously described the synergistic combination of combining MEKi and anti-PD1 [39]. We found that MEKi acted directly on both tumor cells and immune cells globally by reducing PDL1 expression on patient samples and, at low doses, restored some proliferative function of AML-derived T cells. These data provide evidence that treatment strategies that target both tumor-intrinsic factors, as well as T cell suppression, will be more effective.

### Epigenetic regulation of T cell exhaustion

T cell exhaustion is driven by chronic antigen exposure and is accompanied by vast changes in the epigenetic landscape [15, 16, 50–52]. This T cell state is identifiable by three main traits: (1) increased expression of inhibitory receptors such as PD1, LAG3, TIGIT, and CTLA-4, (2) decreased secretion of effector cytokines such as IL-2, IFN-γ, TNF-α, and (3) loss of proliferative capacity [16, 53–56]. Epigenetic changes are largely driven by a pool of coordinating TCR-responsive transcription factors, such as BATF, IRF4, TOX, and NFAT [8, 54–56], chromatin remodeling complexes such as EZH2, and other polycomb repressive complex 2 proteins [57–60]. Generation of TPEx cells relies on the de-repression of many critical pro-memory transcription factors such as TCF1, FOX01, and others as a consequence of targeted DNA methylation deposition acquired during early effector differentiation. Interestingly, the pro-exhaustion transcription factor TOX was found to also directly modulate histone acetylation via direct binding to histone acetyltransferase KAT7 [16, 50, 61]. This critical role for epigenetic regulation of T cell exhaustion has garnered much interest in investigating the potential of several epigenetic targeting SMI, whose original design was directed towards targeting tumor-intrinsic epigenetic dysregulation [14]. Very recently, Milner et al. showed that BRD4 regulates T cell differentiation by promoting super-enhancer activity at regions regulating key pro-exhaustion/differentiation genes such as *Id2*, *Cx3cr1*, and *Runx1* [62]. Using our unique genetically engineered mouse model of AML, we find that BET inhibition in combination with ICB therapy can lead to a shift from predominantly non-responsive T cells with a TEx phenotype to TPEx and the production of functional CD8^+^ T cells. These results form the basis for further study of such combination therapies in AML. In addition, our s3-ATAC-seq data suggest that BETi act directly on TEx cells and enhance their functionality by affecting progenitor program genes accessibility. Our data support the potential for combining BETi with anti-PD1 to rescue anti-PD1 refractoriness in AML and describe a unique role of BRD4 and BRD4 targeting therapies in regulating T cell differentiation. Further studies will focus on understanding the heterogeneity in AML patient responses to BETi + anti-PD1, deeper characterization of the cell specificity of BETi, and the exact mechanism of action by BETi.

## Supplementary information

Supplementary Figure Legends

Supplementary Figure 1

Supplementary Figure 2

Supplementary Figure 3

Supplementary Figure 4

Supplementary Figure 5

Supplementary Figure 6

## Data Availability

S3-ATAC data are publicly available at GSE205368. Additional patient sample clinical information is available at http://www.vizome.org/.
