# Supplementary material for: BET inhibitors rescue anti-PD1 resistance by enhancing TCF7 accessibility in leukemia-derived terminally exhausted CD8+ T cells
Source: Leukemia. 2023 Jan 21;37(3):580–92. doi: 10.1038/s41375-023-01808-0 (PMC9991923; doi:10.1038/s41375-023-01808-0)
Supplement: Supplementary file 1 — Supplementary Figure Legends [file 41375_2023_1808_MOESM1_ESM.docx]

**Supplemental Figures**

**Supplemental Figure 1. Treg, MDSC, and total T cell frequencies in AML and WT mice**

a-c.) Splenocytes isolated from *Flt3-ITD +/-, Tet2 +/-, LysCre +/-* (AML, red) or C57BL/6 (WT, black) mice were isolated and stained for a.) Treg cells (CD4^+^, FOXP3^+^), b.) MDSCs (CD11b^+^, GR1^+^) and c.) all T cells (CD3^+^) and evaluated by flow cytometry. Significances are determined by Mann-Whitney t-tests.

d.) The ratio of CD4 to CD8 T cells derived from *Flt3-ITD +/-, Tet2 +/-, LysCre +/-* (AML, red) or C57BL/6 (WT, black) mice were determined by flow cytometry as previously described. Significance was determined by Mann-Whitney t-test.

**Supplemental Figure 2. CD4^+^ T cell proliferation with BETi + anti-PD1**

1. Plots below are a representative example from an AML mouse demonstrating the gating scheme used to assess T cell phenotype and proliferation.
2. Splenocytes were isolated from 6 AML mice were cultured for 72 hours without TCR stimulation (HIgG), anti-CD3 alone, anti-CD3 with anti-PD1 and titrations of anti-CD3 with JQ1 or anti-CD3 with both JQ1 and anti-PD1. Cells were stained for assessment by flow cytometry. Plots represent the fold-change in proliferation in CD4^+^ T cells, as measured by percent CFSE diluted relative to anti-CD3 stimulated alone. Significance determined by Kruskal-Wallis multiple comparisons t-tests.
3. Fresh mononuclear cells from bone marrow aspirates or peripheral blood obtained from 5 AML patients were stained with CTV and cultured for 5 days without TCR stimulation (mIgG), anti-CD3, anti-CD3 with anti-PD1, anti-CD3 with 120 nM JQ1, or anti-CD3 with 120 nM JQ1 plus anti-PD1. Cells were then stained for assessment by flow cytometry. Plots represent CD4^+^ T cell proliferation for each patient sample. Corresponding patient sample mutations listed in table below.

**Supplemental Figure 3. Other Immune Population vs. *in vivo* BETi + anti-PD1 treatment**

a., b.) Splenocytes derived from AML mice treated with RIgG, JQ1, JQ1 + aPD1, or anti-PD1 were assessed by flow cytometry to determine a.) %MDSCs (CD11b^+^GR1^+^) and b.) %Tregs (CD4^+^FOXP3^+^). No significance was observed between treatments in AML or WT mice independently.

c.-e.) Splenocytes derived from AML mice treated with RIgG, JQ1, JQ1 + aPD1, or anti-PD1 were assessed by flow cytometry to determine c.) %CD8 Naïve T cells (CD62L+, CD44-), d.) %CD8 Central Memory T cells (CD62L^+^CD44^+^), and e.) %CD8 Effector T cells (CD44+, CD62L-). No significance was observed between treatments in AML or WT mice independently. Error bars represent the standard error margin.

**Supplemental Figure 4. QC Metrics for S3-ATAC seq**

a.-b.) QC metrics for the S3-ATAC dataset. a.) Dot plot with the log10 Unique Fragments on the X axis vs median TSS on the Y-axis. A minimum TSS threshold of 8 was used for downstream analyses. b.) plots the %fragments vs basepair size of fragments for the dataset. Plots generated in ArchR.

**Supplemental Figure 5. S3-ATAC marker gene scores vs cluster**

1. Gene scores with imputation were calculated with ArchR and visualized across the UMAP clustering. Genes relating to T cell subtype (row 1) are listed from left to right – CD3, CD4, CD8, SELL, T cell exhaustion (row 2) – PDCD1 (encoding PD1), HAVCR2 (encoding TIM3), TCF7 (encoding TCF1), and TOX, and related myeloid and Treg markers (row 3) – CD14, LAG3, FOXP3, and CXCR3.

**Supplemental Figure 6. JQ1 induces changes in gene accessibility through Tex differentiation**

a-b.) Gene activity score matrices were calculated in ArchR for cells throughout the Tex differentiation trajectory for a.) Vehicle and b.) JQ1 treated cells. Plots created with ArchR.
