# Supplementary figures and images for: BET inhibitors rescue anti-PD1 resistance by enhancing TCF7 accessibility in leukemia-derived terminally exhausted CD8+ T cells
Source: Leukemia. 2023 Jan 21;37(3):580–92. doi: 10.1038/s41375-023-01808-0 (PMC9991923; doi:10.1038/s41375-023-01808-0)

Figure S1

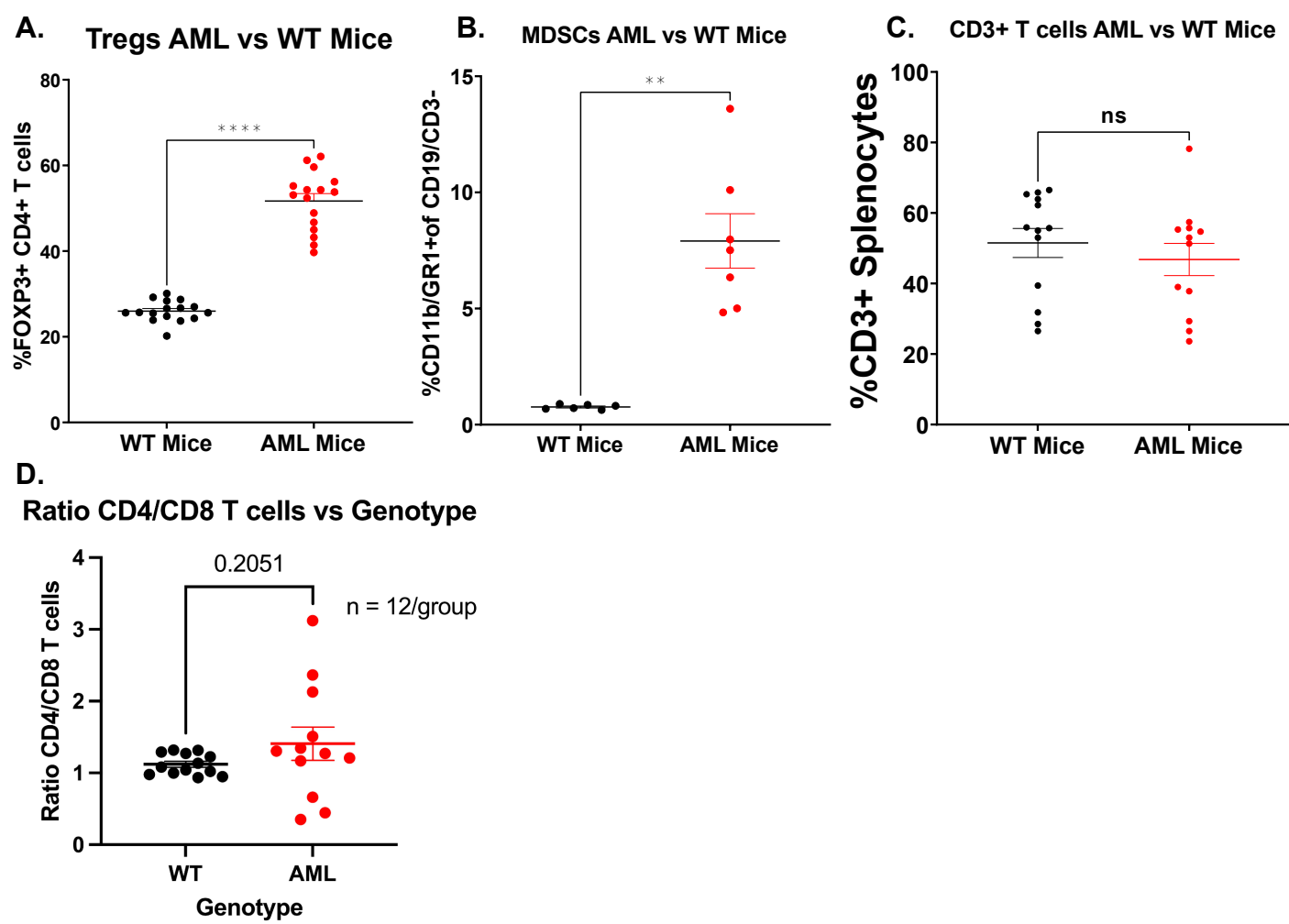

Supplement: Supplementary file 2 — Supplementary Figure 1 [file 41375_2023_1808_MOESM2_ESM.pdf]

Figure S3

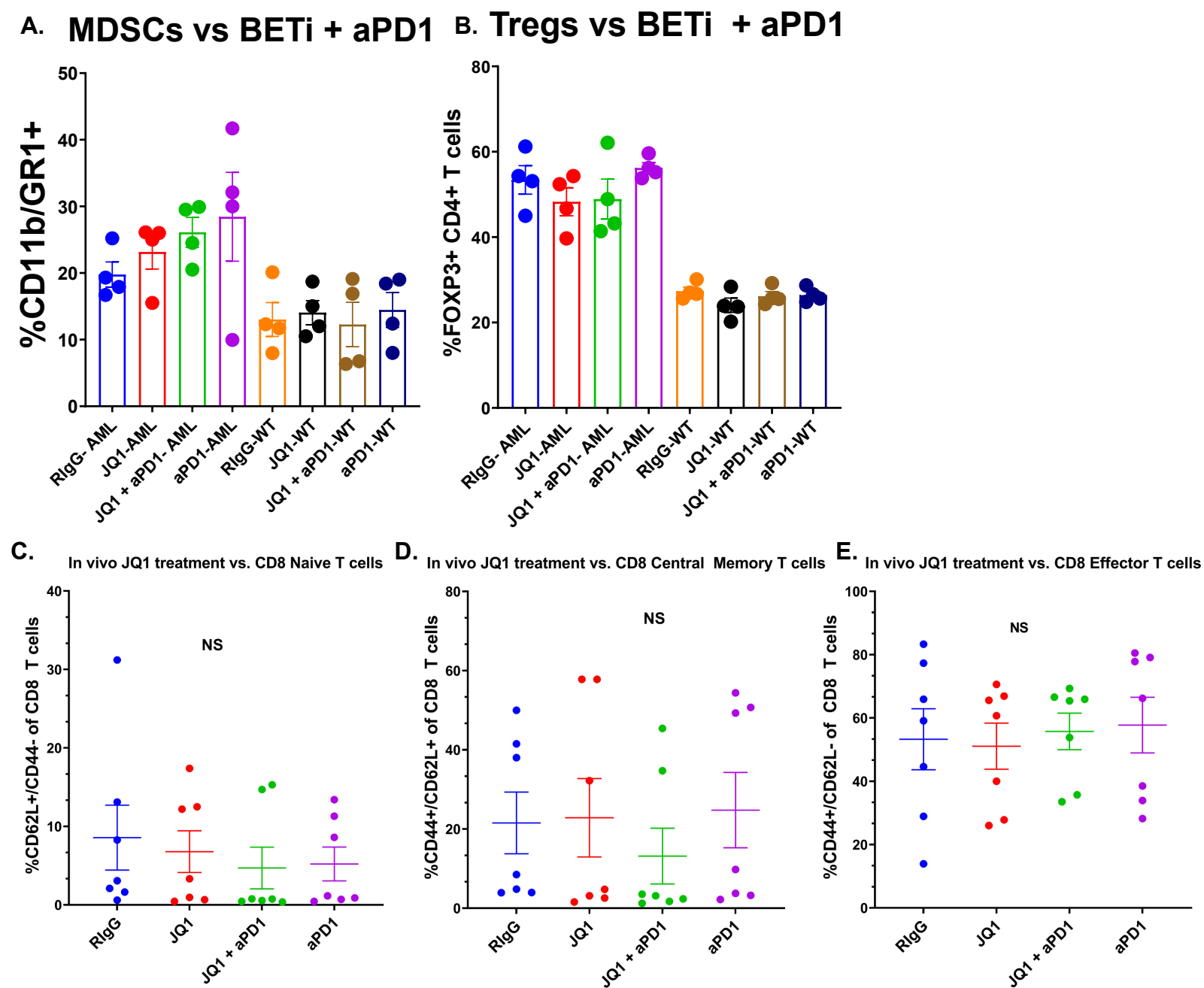

Supplement: Supplementary file 4 — Supplementary Figure 3 [file 41375_2023_1808_MOESM4_ESM.pdf]

Figure S6

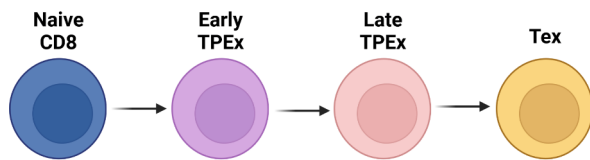

A.

Vehicle

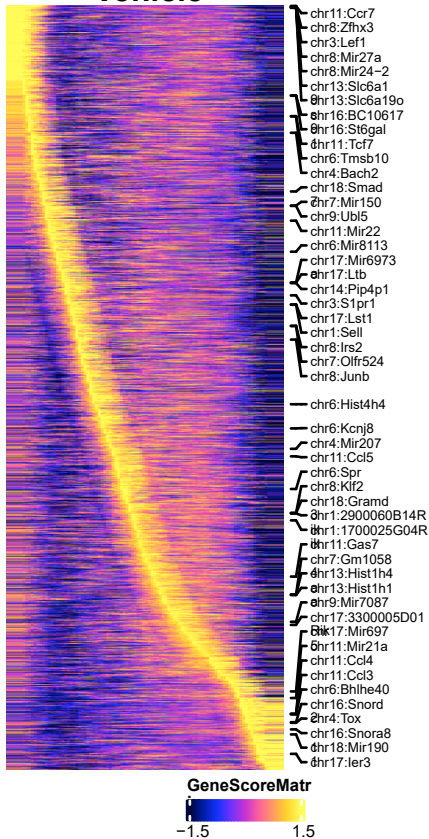

B.

JQ1

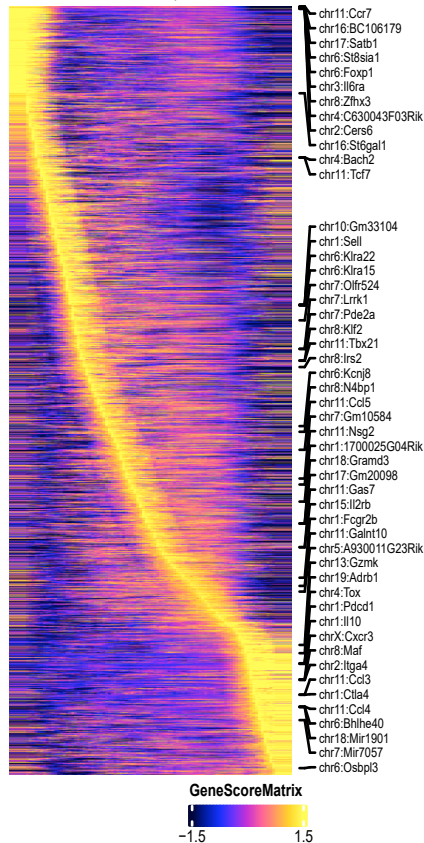

Supplement: Supplementary file 7 — Supplementary Figure 6 [file 41375_2023_1808_MOESM7_ESM.pdf]
