# Supplementary material for: BET inhibitors rescue anti-PD1 resistance by enhancing TCF7 accessibility in leukemia-derived terminally exhausted CD8+ T cells
Source: Leukemia. 2023 Jan 21;37(3):580–92. doi: 10.1038/s41375-023-01808-0 (PMC9991923; doi:10.1038/s41375-023-01808-0)
Supplement: Supplementary file 3 — Supplementary Figure 2 [file 41375_2023_1808_MOESM3_ESM.pdf]

Figure S2

A.

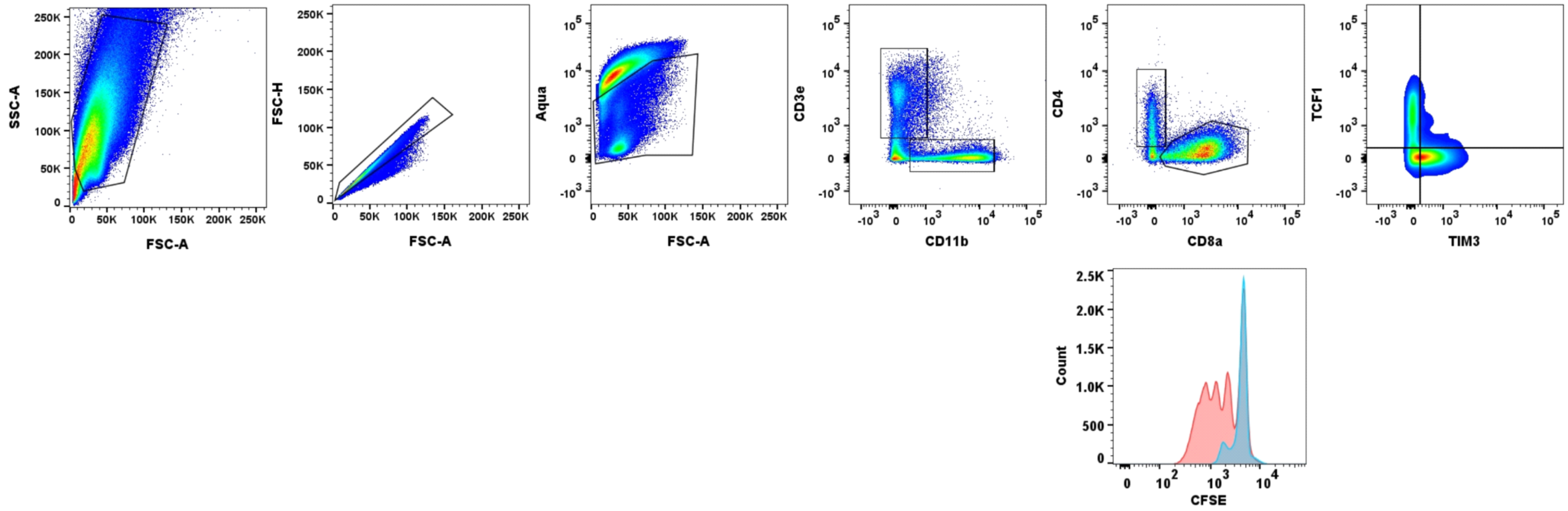

B.

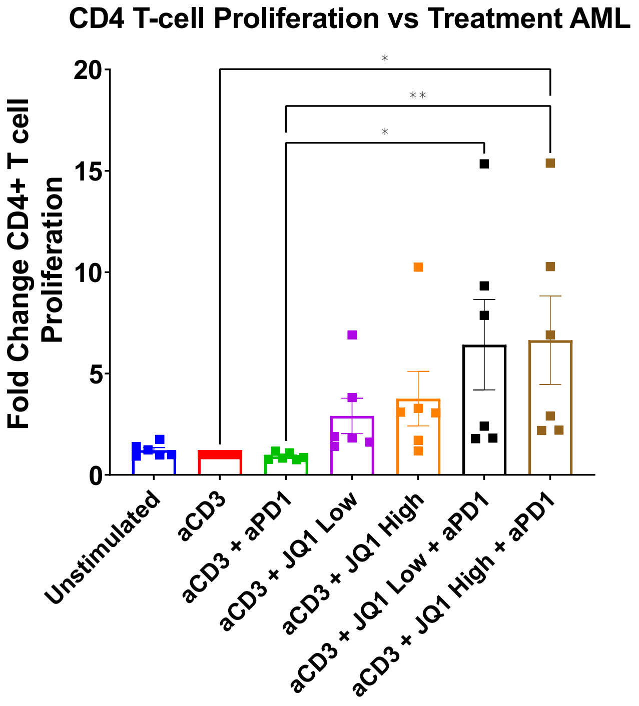

C.

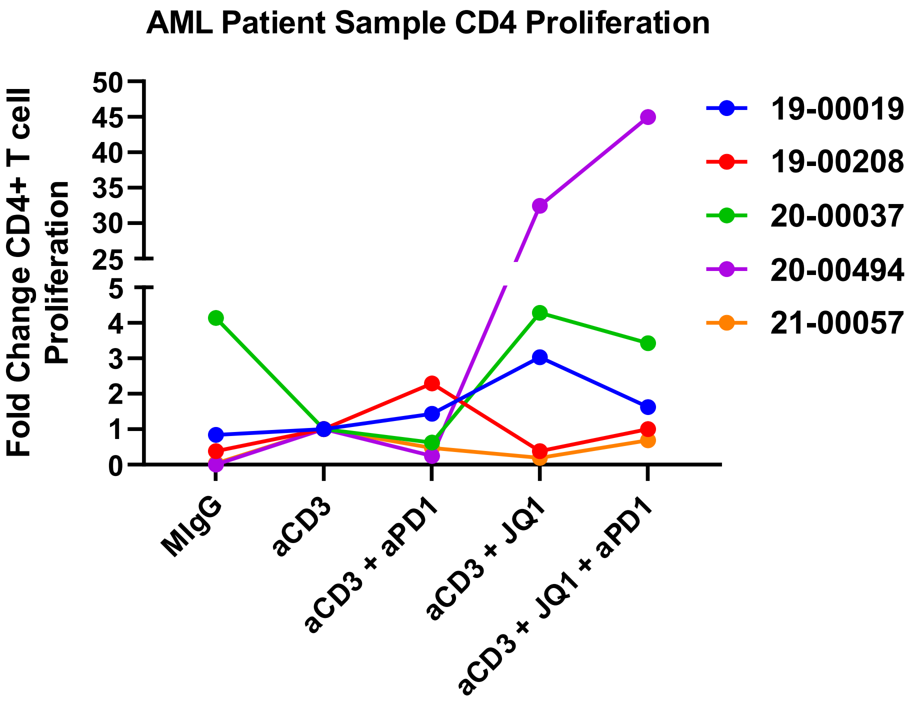

Pt-ID	Mutations						
19-00019	CEBPA	DNMT3A	GATA2	NRAS			
19-00208	FLT3-ITD						
20-00037	CEBPA	DNAH9	DOCK8	EGFR	EZH2	GATA2	NPM1
21-00494	DNMT3A	IDH2	NT5CS2	RUNX1	ASXL1	BCOR	
21-00057	SMC1A	PTPN11	NPM1	DNMT3A	IDH1		
22-00394	CEBPA	ASXL1	WT1	IKZF1	PTPN11	DNM2	UBR5
22-00424	DNMT3A	NPM1	GATA2	NRAS	CHEK2	IRF4	FAT4
