# Supplementary material for: BET inhibitors rescue anti-PD1 resistance by enhancing TCF7 accessibility in leukemia-derived terminally exhausted CD8+ T cells
Source: Leukemia. 2023 Jan 21;37(3):580–92. doi: 10.1038/s41375-023-01808-0 (PMC9991923; doi:10.1038/s41375-023-01808-0)
Supplement: Supplementary file 5 — Supplementary Figure 4 [file 41375_2023_1808_MOESM5_ESM.pdf]

Figure S4

A.

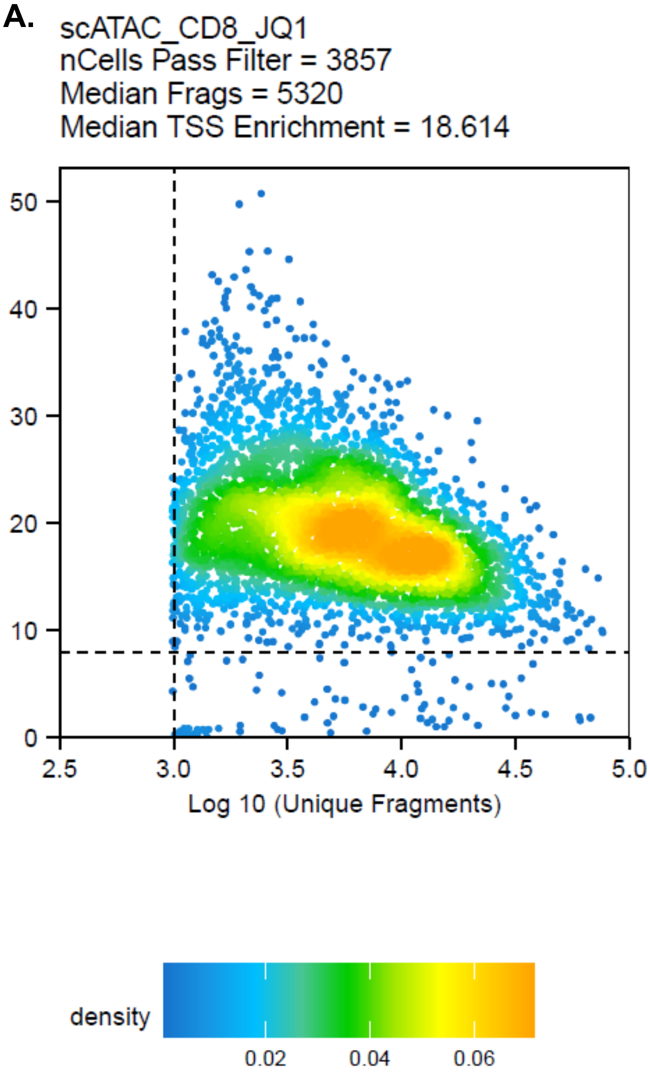
scACTAC_CD8_JQ1
nCells Pass Filter = 3857
Median Frags = 5320
Median TSS Enrichment = 18.614
Log 10 (Unique Fragments)
density
0.02
0.04
0.06

B.

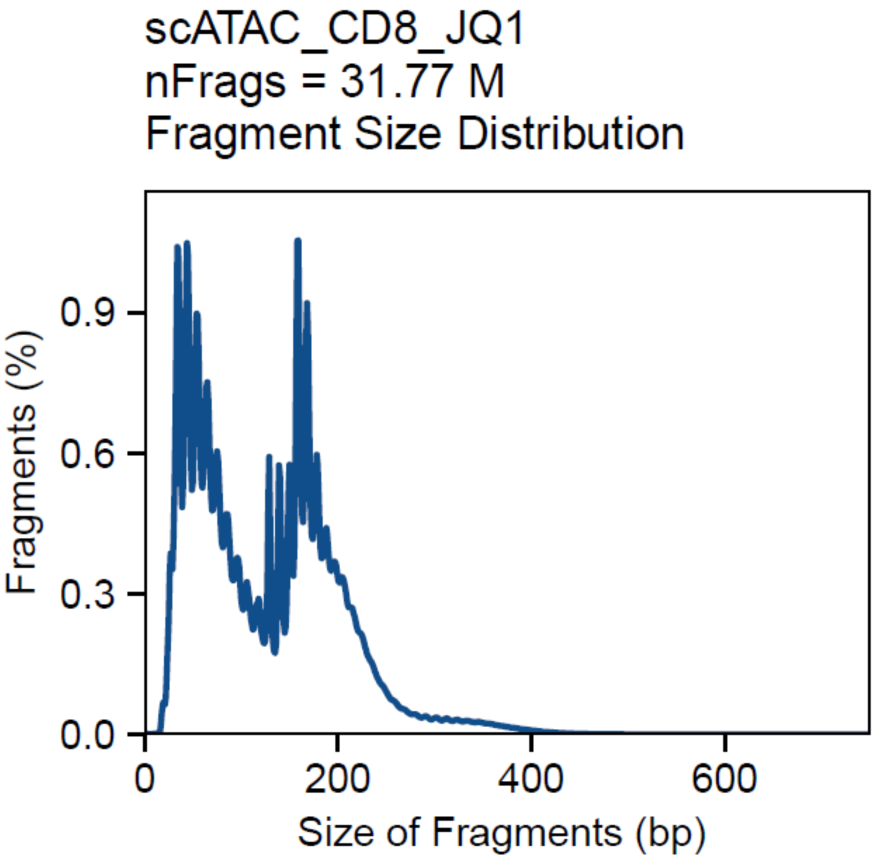
scATAC_CD8_JQ1
nFrags = 31.77 M
Fragment Size Distribution
Fragments (%)
Size of Fragments (bp)
