# Supplementary material for: BET inhibitors rescue anti-PD1 resistance by enhancing TCF7 accessibility in leukemia-derived terminally exhausted CD8+ T cells
Source: Leukemia. 2023 Jan 21;37(3):580–92. doi: 10.1038/s41375-023-01808-0 (PMC9991923; doi:10.1038/s41375-023-01808-0)
Supplement: Supplementary file 6 — Supplementary Figure 5 [file 41375_2023_1808_MOESM6_ESM.pdf]

Figure S5

A.

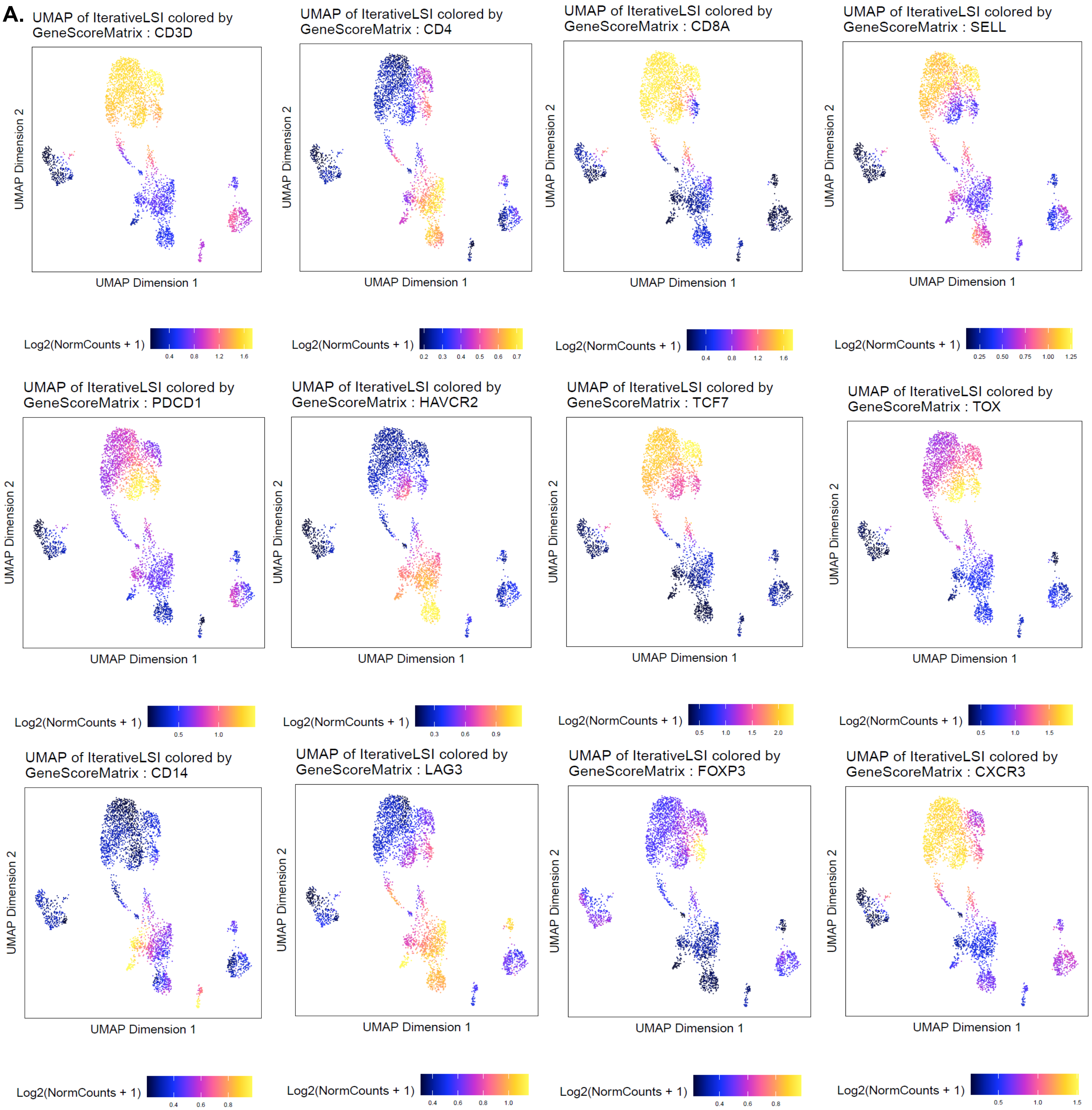
UMAP of IterativeLSI colored by GeneScoreMatrix : CD3D
UMAP Dimension 2
UMAP Dimension 1
Log2(NormCounts + 1)
0.4 0.8 1.2 1.6
UMAP of IterativeLSI colored by GeneScoreMatrix : CD4
UMAP Dimension 2
UMAP Dimension 1
Log2(NormCounts + 1)
0.2 0.3 0.4 0.5 0.6 0.7
UMAP of IterativeLSI colored by GeneScoreMatrix : CD8A
UMAP Dimension 2
UMAP Dimension 1
Log2(NormCounts + 1)
0.4 0.8 1.2 1.6
UMAP of IterativeLSI colored by GeneScoreMatrix : SELL
UMAP Dimension 2
UMAP Dimension 1
Log2(NormCounts + 1)
0.25 0.50 0.75 1.00 1.25
UMAP of IterativeLSI colored by GeneScoreMatrix : PDCD1
UMAP Dimension 2
UMAP Dimension 1
Log2(NormCounts + 1)
0.5 1.0
UMAP of IterativeLSI colored by GeneScoreMatrix : HAVCR2
UMAP Dimension 2
UMAP Dimension 1
Log2(NormCounts + 1)
0.3 0.6 0.9
UMAP of IterativeLSI colored by GeneScoreMatrix : TCF7
UMAP Dimension 2
UMAP Dimension 1
Log2(NormCounts + 1)
0.5 1.0 1.5 2.0
UMAP of IterativeLSI colored by GeneScoreMatrix : TOX
UMAP Dimension 2
UMAP Dimension 1
Log2(NormCounts + 1)
0.5 1.0 1.5
UMAP of IterativeLSI colored by GeneScoreMatrix : CD14
UMAP Dimension 2
UMAP Dimension 1
Log2(NormCounts + 1)
0.4 0.6 0.8
UMAP of IterativeLSI colored by GeneScoreMatrix : LAG3
UMAP Dimension 2
UMAP Dimension 1
Log2(NormCounts + 1)
0.4 0.6 0.8 1.0
UMAP of IterativeLSI colored by GeneScoreMatrix : FOXP3
UMAP Dimension 2
UMAP Dimension 1
Log2(NormCounts + 1)
0.4 0.6 0.8
UMAP of IterativeLSI colored by GeneScoreMatrix : CXCR3
UMAP Dimension 2
UMAP Dimension 1
Log2(NormCounts + 1)
0.5 1.0 1.5
